# Hepatic arterial infusion chemotherapy in hepatocellular carcinoma: A bibliometric and knowledge-map analysis

**DOI:** 10.3389/fonc.2022.1071860

**Published:** 2023-01-04

**Authors:** Mei Li, Kai Zhang, Ji’an He, Weihao Zhang, Tianye Lv, Li Wang, Wenge Xing, Haipeng Yu

**Affiliations:** ^1^ Department of Interventional Therapy, Tianjin Medical University Cancer Institute & Hospital, National Clinical Research Center for Cancer, Tianjin, China; ^2^ Tianjin’s Clinical Research Center for Cancer, Tianjin Medical University Cancer Institute & Hospital, Tianjin, China; ^3^ Key Laboratory of Cancer Prevention and Therapy, Tianjin Medical University Cancer Institute & Hospital, Tianjin, China

**Keywords:** hepatocellular carcinoma, bibliometric, hepatic arterial infusion chemotherapy, data mining, FOLFOX

## Abstract

**Background:**

In recent years, hepatic arterial infusion chemotherapy (HAIC) has gained popularity in the treatment of hepatocellular carcinoma. Although several studies have been published, no bibliometric analysis have been conducted on this topic.

**Objectives:**

To understand the development status and future trends in the application of HAIC, we conducted bibliometric analysis to examine the cooperation and influence among countries, institutions, authors, and journals.

**Methods:**

All relevant articles and reviews on the use of HAIC in HCC treatment were retrieved from the Web of Science database. A bibliometric analysis of countries, institutions, journals, authors, and keywords related to this field was performed using R and VOSviewer software. The main aspects analyzed were the research status and key fields of HAIC in HCC treatment.

**Results:**

A total of 1026 articles published in 292 journals by 4937 authors from 959 institutions between 1974 and 2021 were retrieved. A rapid increase in articles published after 1990 was observed, which reached the peak in 2021. Japan had the most publications and citations. Yonsei University, Sun Yat-sen University, and Hiroshima University were the three leading institutions in research on this topic. Kwang-Hyub Han and Masatoshi Kudo have the greatest academic influence in this field. Most publications were made in the Hepato-Gastroenterology, whereas cancer had the most citations. The main aspects of HAIC treatment of HCC include HAIC and TACE, chemotherapy drug selection, HAIC and targeted therapy and immunotherapy, HAIC and surgery, and hepatotoxicity. Keywords such as FOLFOX, lenvatinib, hepatic arterial infusion chemotherapy are hot words in this field in recent years.

**Conclusion:**

The research on the use of HAIC in the treatment of HCC has been on the rise. Currently, HAIC combined with targeted therapy or immunotherapy has attracted significant attention.

## Introduction

Globally, hepatocellular carcinoma (HCC) is the sixth most common cancer and the third leading cause of cancer-related death (1, 2). For HCC patients, surgery is the most effective treatment. However, most newly diagnosed HCCs are in the middle and late stages and cannot be surgically resected (3, 4). For such patient, the prognosis can only be improved by application of local or systemic therapy. In Asia, hepatic arterial infusion chemotherapy (HAIC) is a common local treatment for unresectable liver cancer (5, 6).

Vascular intervention through an intra-arterial catheter introduces chemotherapeutic drugs used in HAIC to kill tumor cells. As a form of local chemotherapy, HAIC has several advantages over systemic intravenous chemotherapy (7, 8). First, HAIC delivers a high concentration of drugs continuously to the tumor through arteries in it, avoiding the “first-pass effect.” In addition, HAIC can kill tumors while reducing systemic side effects (9–11).

Studies have demonstrated that HAIC can improve the prognosis of patients with middle to advanced liver cancer (12–15). Over the past years, there has been an increase in research on the use of HAIC in the treatment of HCC. However, there is no clear direction in the latest research progress and hotspots in this area. Therefore, there is a need to summarize the global research trends and hotspots in the field. Unfortunately, to the best of our knowledge, no bibliometric analysis has been reported for this area.

Bibliometric analysis summarizes data and publication characteristics in a certain field based on the available qualitative data (16, 17). The data is analyzed to understand the knowledge structure and identify research fronts or hot spots in a given field. It also evaluates global scientific publications and reveal the latest progress and trends over time in a given field (18).

There are several bibliometric articles on hepatocellular carcinoma (19–22). However, there is no bibliometric articles on the use of HAIC for HCC treatment. Herein, we summarize the application and developmental trends in the application of HAIC in HCC treatment through bibliometric analysis of available data. The findings of this research reveal research progress, hotspots, and development trends in the field. This analysis reveals the future research direction in this area.

## Methods

### Database

Relevant articles were retrieved from the Web of Science Core Collection (WoSCC) database. The WoSCC was selected because it provides comprehensive data for bibliometric software requirements and is one of the most influential databases for this type of study (23–26). Ethical approval was not required for this study since the data were downloaded directly from public databases.

### Search criteria

The WoSCC database was searched for all relevant literature on HAIC application in the treatment of HCC up to December 2021. The retrieved papers were exported as “full-text records and references” and saved in “plain text” format. The specific search terms used are listed in **Supplementary Figure S1**. The downloaded file was filtered using the bibliometrix package in the R 4.1.3 software to retain only articles and reviews in English published from January 1974 to December 2021.

### Data analysis and visualization

Automated data analysis and visualization were conducted using bibliometrix. Bibliometrix is an open-source tool that performs comprehensive science mapping analysis of scientific literature (27–29). In this study, it was used to examine the number of publications and collaborations across countries, institutions, authors, and journals (30). The cooperation relationship is mainly shown using a cooperation network diagram. In the cooperative network graph, the size of nodes represents centrality, the line represents co-occurrence relationship, and the same color of nodes represents the same cluster. The Bibliometrix package can evaluate an author’s influence using the h-index, g-index, or m-index. The h-index is a mixed indicator that evaluates the level and quantity of academic output based on the h articles published by an author that have been cited no less than h times (31, 32). The g-index compensates where the h-index cannot reflect highly cited articles well, making it an ideal supplement to the h-index. The m-index is an h-index calculated based on academic age. Furthermore, the number of times an article is cited may reflect how it disseminates information, its influence on the field, and the overall quality and significance. The Bibliometrix package’s trending topics feature reveals trends in abstract words.

VOSviewer is a commonly used visualization and construction software tool for bibliometric papers. Keyword cluster analysis and visualization were conducted using VOSviewer 1.6.18 (33, 34). All keywords occurring more than 10 times from the articles were extracted using the VOSviewer software. Finally, 167 keywords were extracted and divided into five clusters.

The collaborative and cluster analysis graphs from the above software were based on co-authorship and co-occurrence (35, 36).

## Results

### The trend in publications and citations

**Figure 1:**
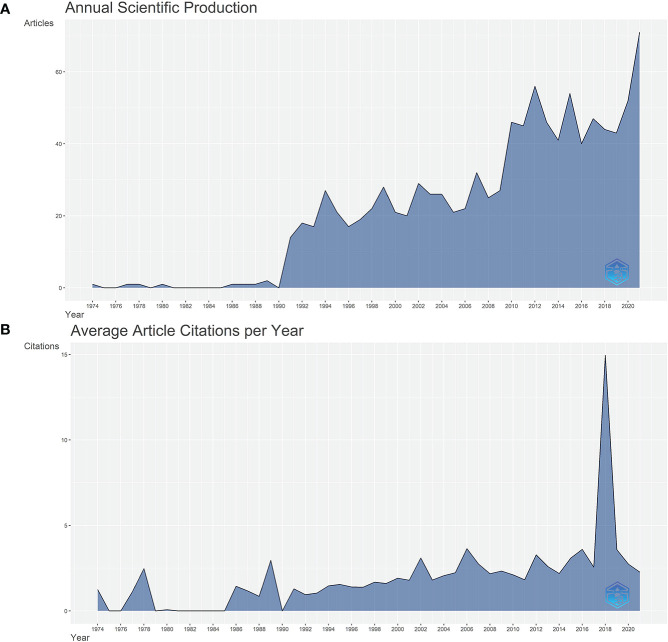
**(A)** A line chart of the number of publications published each year, 1973-2021, as detailed in **Supplementary Figure S3**. **(B)** A line chart showing the average number of citations per article per year. Specific data are in the **Supplementary Material Figure S3**.

A total of 1026 publications, including 952 articles and 84 reviews, were retrieved. The graph shown in **Figure 1A** illustrates the number of publications over the studied period. Before 1990, only one to three articles were published per year. However, the number of publications has been increasing significantly since 1990 on a yearly time-frame. In the past 10 years, research on HAIC therapy in HCC treatment has increased rapidly. A total of 83 studies were published in 2021, which is the highest number of publications over the years. **Figure 1B** shows the average number of citations for publications recorded each year, with the highest number of citations obtained in 2018. This peak indicates there may have been breakthrough publications in 2018, which may explain another sharp increase in publications in 2021.

### Institutional and country contributions

Thirty-one countries have published articles in this field. **Table 1** indicates the top 10 authors based on country, scientific impact, and international collaboration in the application of HAIC in HCC treatment. As shown in **Table 1**, Japan (456 articles) had the highest number of publications, followed by China (197 articles), South Korea (96 articles), and the United States (US) (83 articles). By December 2021, all publications had been cited 17,686 times, with an average of 28.7 citations per article. Japanese publications were the most cited, followed by the US and Chinese publications.

**Table 1:** The top 10 corresponding authors for country, scientific impact, and international collaboration.

Rank	Country	Articles	SCP	MCP	Total citations	Average article citations
1	JAPAN	456	450	6	12814	28.1
2	CHINA	212	197	15	3790	17.88
3	KOREA	101	96	5	3369	33.36
4	USA	99	83	16	3870	39.09
5	ITALY	29	28	1	788	27.17
6	FRANCE	19	18	1	863	45.42
7	GERMANY	18	13	5	486	27.00
8	SPAIN	9	8	1	291	32.33
9	UNITED KINGDOM	7	6	1	924	132.00
10	SWEDEN	6	6	0	40	6.67

SCP, Single Country Publications; MCP, Multiple Country Publications.

**Figure 2:**
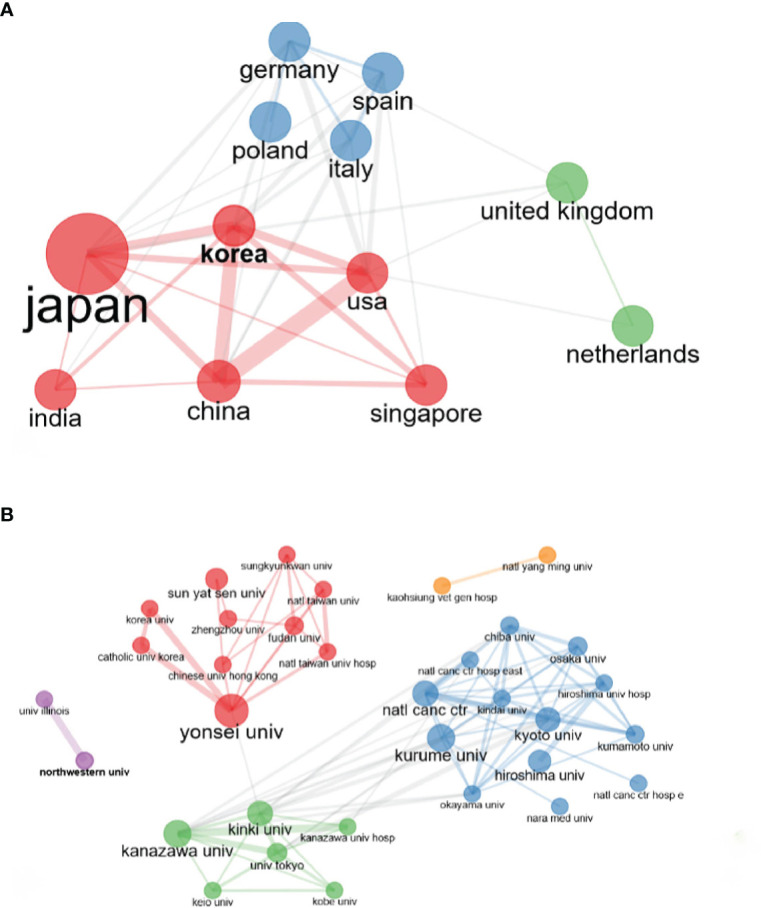
**(A)** This is a collaboration network diagram between countries. The color of the nodes represents clustering and the size of the nodes represents centrality and lines between nodes represent the strength of collaborations. **(B)** This is the collaboration network diagram between organizations. The color of nodes represents clustering and the size of nodes represents centrality and lines between nodes represent the strength of collaborations.

**Figure 2A** shows the collaboration network between countries. In **Figure 2A**, the lines between the nodes in the graph represent the degree of cooperation between countries. The thicker the line, the greater the degree of cooperation. Japan is at the center of the network and has the highest centrality. Japan’s most important partners are the US, China, South Korea, and Singapore. A close collaboration was observed between China and the United States.

Over 959 institutions applied HAIC in HCC treatment. The top 10 institutions with the highest contributions are summarized in **Table 2**. In terms of the number of articles published, Yonsei University (135 articles), Sun Yat-sen University (85 articles), and Hiroshima University (57 articles) were the three most prolific academic institutions. The majority of the top 10 institutions were from Asian countries, including two from China. **Figure 2B** shows the collaboration network diagram between organizations. **Figure 2B** shows a network of cooperative relationships between institutions with different colors representing institutions that have collaborated before. Chinese institutions cooperate more with Korean institutions, while Japanese institutions cooperate more with each other.

**Table 2:** Top 10 institutions with the most articles.

Rank	Affiliations	Country	Articles
1	YONSEI UNIV	Korea	135
2	SUN YAT SEN UNIV	China	85
3	HIROSHIMA UNIV	Japan	57
4	FUDAN UNIV	China	42
5	KURUME UNIV	Japan	42
6	CATHOLIC UNIV KOREA	Korea	39
7	KANAZAWA UNIV	Japan	38
8	SEOUL NATL UNIV	Korea	34
9	KYOTO UNIV	Japan	32
10	NORTHWESTERN UNIV	America	31

### Key authors and co-cited authors

**Figure 3:**
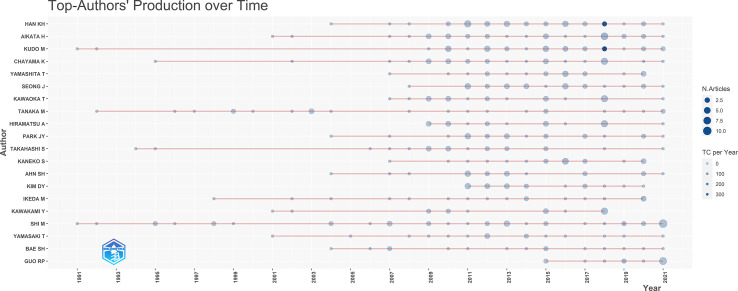
This graph represents top-authors’ production over time, and the node size represents total number of articles. The color of the nodes represents the yearly average number of times each document has been cited.

The 1026 studies included in the present analysis were published by 4937 authors, 8701 of whom are co-cited. **Figure 3A** shows the 10 most prolific authors, with the top three being Han KN, Aikata H, and Kudo M. The nodes represent the number of publications published by the author in a given year. The larger the node, the more publications the author has published, and the darker the node color, the more citations the author received in the year. For most authors, the publication rate has increased over the past 10 years. Han KN and Kudo M published important articles in 2018 with annual citations exceeding 300. **Table 3** shows the academic influence indicators, such as the h-index, g-index, and m-index, for the top 10 authors with the highest number of publications. Kudo M ranked first in the h-index and total citations and second in the g-index. Meanwhile, Han KN ranked first in the g-index and number of most publications and second in the h-index and total citations, demonstrating that the two authors have made important contributions to the field.

**Table 3:** The 10 most productive authors and the top 10 co-cited authors with the highest citations.

Rank	Author	H	G	M	TC	NP
1	HAN KH	17	33	0.895	2743	33
2	AIKATA H	17	27	0.773	773	32
3	KUDO M	21	31	0.656	3337	31
4	CHAYAMA K	17	23	0.607	573	28
5	YAMASHITA T	16	24	1	585	24
6	SEONG J	13	23	0.867	640	23
7	KAWAOKA T	13	20	0.813	433	22
8	TANAKA M	13	21	0.419	850	21
9	HIRAMATSU A	13	19	0.929	379	20
10	PARK JY	14	20	0.737	647	20

NP, Number of Publications; TC, Total Citations.

The studies were published in 292 journals (**Figure S5**). Hepato-Gastroenterology, Hepatology Research, and Journal of Vascular and Interventional Radiology were among the top 3 most preferred journals. Studies in the CANCER journal were the most cited.

### Co-occurrence of keywords

**Figure 4:**
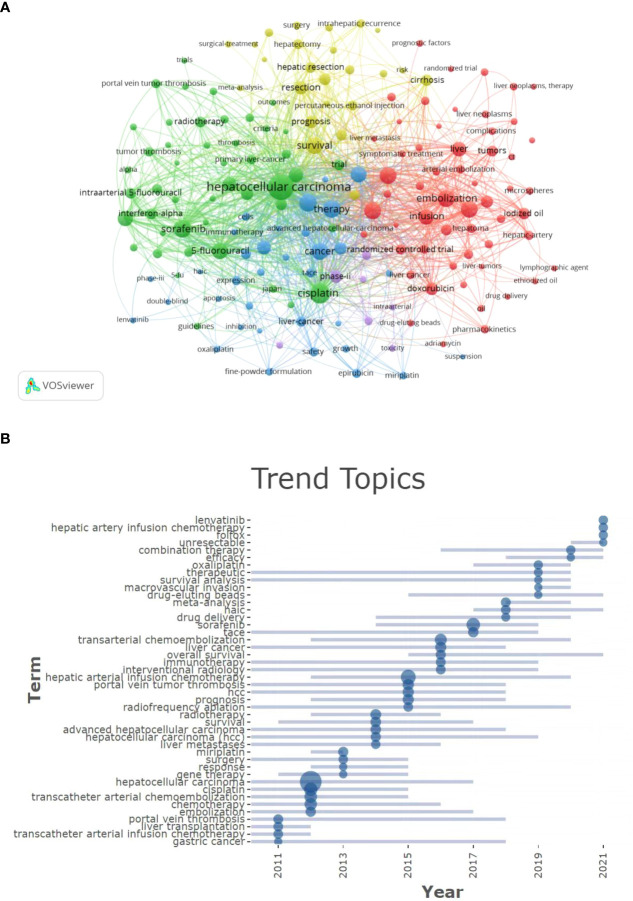
**(A)** This is a keyword co-occurrence analysis cluster map. Each color represents a cluster. **(B)** The graph shows the change over time of the most frequent keywords in each year, with the size of the blue nodes representing the frequency of keyword occurrences. A maximum of 5 keywords are displayed per year.The whole picture is in **Supplementary Material Figure S4**.

Potential research hotspots were identified by analyzing the co-occurrences of keywords. A total of 3037 keywords were extracted using the VOSviewer software, among which 167 appeared more than ten times. **Table 4** shows that the most frequently used keyword was hepatocellular carcinoma, followed by cisplatin and transarterial chemoembolization. The keywords were divided into five clusters based on their link strength (**Figure 4A**). Each color represents a cluster, while the node’s size represents the keyword’s frequency. The largest cluster (red) had 56 co-occurring words related to HAIC and transcatheter arterial chemoembolization (TACE). They include embolization, chemoembolization, chemotherapy, doxorubicin, hepatocellular carcinoma, infusion, lipiodol, transcatheter arterial embolization, etc. Cluster 2 (green) had 46 terms related to drug research used, including hepatocellular carcinoma, sorafenib, cisplatin, 5-fluorouracil, arterial infusion chemotherapy, interferon-alpha, etc. The articles in cluster 3 (blue) mainly discuss HAIC with targeted therapy or immunotherapy. Among the 34 keywords in the cluster, the most prominent were infusion chemotherapy, immunotherapy, angiogenesis, efficacy, expression, lenvatinib, safety, growth, and inhibition. Cluster 4 (yellow) had 24 co-occurring words related to HAIC and surgery research: survival, ablation, arterial infusion, surgery, resection, curative resection, hepatic resection, recurrence, prognosis, risk factors, etc. Cluster 5 (purple) had seven co-occurring words related to drug hepatotoxicity research: carcinoma, hepatocellular, intra-arterial chemotherapy, intraarterial, cirrhosis, phase-ii, and toxicity.

**Table 4:** The top 20 most frequently used keywords.

Rank	Keywords	Occurrences	TLS
1	hepatocellular carcinoma	473	3190
2	cisplatin	230	1838
3	transarterial chemoembolization	180	1351
4	therapy	172	1275
5	sorafenib	170	1319
6	chemotherapy	163	1079
7	chemoembolization	161	1126
8	embolization	160	1161
9	cancer	150	1010
10	survival	149	1140
11	hepatocellular-carcinoma	148	680
12	transcatheter arterial chemoembolization	142	1086
13	5-fluorouracil	135	1061
14	infusion	125	845
15	hepatic arterial infusion chemotherapy	119	875
16	liver	99	599
17	management	99	788
18	arterial infusion chemotherapy	95	615
19	efficacy	91	735
20	resection	83	591

TLS, total link strength.

**Figure 4B** illustrates how keywords have changed over time. Blue nodes represent the most common keywords in a given year, and their size indicates their frequency. These keywords are regarded as research hotspots for the given year. FOLFOX, lenvatinib, hepatic arterial infusion chemotherapy, undetectable, curative effects, combined therapies, among others are the research hotspots which have appeared frequently in the past three years and may remain so for a long time.

## Discussion

In this age of big data, researchers in a given field aim to understand the development trends of the field. The bibliometric analysis can use several bibliometric software tools to analyze and visualize existing literature comprehensively (37, 38).

Multiple studies have been conducted to investigate the use of HAIC in HCC treatment in the past few decades. The application of HAIC in HCC treatment was first proposed in the 1990s by Japanese scholars, and studies in this area have risen ever since. The number of articles published in the past 10 years has increased rapidly, reaching the peak in 2021.

Japan has made tremendous contributions to HAIC and HCC-related research. Other notable nations with many HAIC and HCC-related studies include South Korea, China, and the US. Asian countries have published more studies on HAIC than European countries because of the high prevalence of viral hepatitis and HCC in this continent (2, 39, 40). Despite starting late, China has become one of the largest contributors in this field, which may be due to China’s economic development and increase in academic research funding. However, China has an average of 17.88 citations per article, which is lower than the average (28.7). Notably, 4 of the top 10 institutions with significant contributions in this field are in Japan, making it the top most influential and advanced countries in the world in this area. According to the institutional cooperation analysis results, international institutions generally do not collaborate in research on HAIC for HCC treatment. We believe that close collaboration among countries will enhance research on this topic.

The indices of Han KN and Kudo were very high, and their findings have contributed immensely to the rapid development of HAIC application. The annual citations of their articles published in 2018 exceeded 300. Two of their most cited articles are “Lenvatinib versus sorafenib in first-line treatment of patients with unresectable hepatocellular carcinoma: a randomized phase 3 non-inferiority trial” and “Sorafenib plus low-dose cisplatin and fluorouracil hepatic arterial infusion chemotherapy versus sorafenib alone in patients with advanced hepatocellular carcinoma (SILIUS): a randomized, open-label, phase 3 trial.” REFLECT showed that the efficacy of lenvatinib in treating HCC was comparable to that of sorafenib. This was the first phase 3 clinical study to show positive results in the first-line treatment of advanced liver cancer, making lenvatinib the first drug identified to have comparable efficacy to sorafenib (41). SILIUS confirmed that sorafenib combined with HAIC had higher ORR and longer TTP than sorafenib alone, but overall survival was similar in both groups (42).

Most articles were published in the “Hepato-Gastroenterology” journal. Despite publishing fewer articles, articles in the Lancet journal were the most cited, making it the most influential journal, as evidenced by its high impact factor of 202.731. The other journals with many publications were “Journal of Vascular and Interventional Radiology,” “Cardiovascular and Interventional Radiology”, and “Cancer”.

The co-occurrence of keywords in bibliometrics can reflect academic hotspots. Based on cluster analysis, five main aspects of HAIC treatment of HCC were identified, including HAIC and TACE, chemotherapy drug selection, HAIC and targeted therapy and immunotherapy, HAIC and surgery, and hepatotoxicity.

HAIC and TACE are the major types of vascular interventional therapy for liver cancer. Transcatheter arterial chemoembolization is the most commonly used method for treating mid-stage HCC (43). However, patients with mid-stage liver cancer are a heterogeneous population, and the efficacy of TACE depends largely on the tumor burden (44). Chemotherapeutic drugs can be continuously delivered locally in the tumor-feeding artery through HAIC without using embolic agents, suggesting that HAIC is a promising therapeutic approach for the treatment of large HCC (45, 46). Researchers have found that HAIC may have even greater outcomes than TACE in treating advanced HCC within a stage suitable for TACE (47, 48).

Currently, there is no standard chemotherapy regimen for HAIC, and the choice of drugs differ in different regions, which may explain the different efficacies reported in the available literature. The most commonly used chemotherapy drugs in Japan are cisplatin and 5-FU. Other commonly used chemotherapy drugs include oxaliplatin, carboplatin, epirubicin, and etoposide. In EACH, a phase III clinical trial, FOLFOX provided better results than doxorubicin in patients with advanced HCC (49). FOLFOX chemotherapy was first proposed by Chinese researchers, and further research has demonstrated that the FOLFOX chemotherapy regimen is an effective treatment for HCC (50–52).

With the advent of targeted and immune drugs, there is need to compare the efficacy of a combination of targeted therapy or immunotherapy with HAIC (53). This is currently a research hotspot in the field of HAIC. Several studies have shown that HAIC is more effective when combined with targeted immunotherapy (54–57)

Surgery is the primary method for treating liver cancer (58, 59). However, surgical resection is no longer recommended for patients with large and multiple tumors or vascular metastases. HAIC is mainly used for treating unresectable liver cancer. In the 2021 ASCO annual meeting, researchers from Sun Yat-Sen University reported that preoperative FOLFOX-HAIC neoadjuvant therapy can improve the outcome of resected HCC patients with ultra-Milan standard BCLC stage A/B (60). Another study showed that patients with initially unresectable HCC have an extremely high surgical conversion rate when treated with targeted therapy plus immunotherapy plus HAIC (61).

## Limitations

Regarding limitations, firstly, our study only analyzed articles in the WoSCC published in English. According, important publications could have been omitted. Nevertheless, WoSCC is a commonly used database for bibliometric analysis that captures most of the information. Secondly, since it takes time for an article to be cited, recently published high-quality articles might still be in this window and, thus, left out.

## Conclusion

In conclusion, HAIC has increasingly become a popular treatment for HCC. Japan has been and continues to be at the forefront of related research. The main point of this study is that future studies should focus on the efficacy of HAIC in combination with targeted therapy and immunotherapy in patients with HCC.

## Data availability statement

Publicly available datasets were analyzed in this study. This data can be found here: Web of Science Core Collection.

## Author contributions

ML: data collection and drafting of the manuscript, KZ: drafting and revision, JH: data analysis, WZ: data collection, TL and LW: data collection, WX and HY: design of this work and data analysis. All authors contributed to the article and approved the submitted version.
