# Supplementary material for: Hepatic arterial infusion chemotherapy in hepatocellular carcinoma: A bibliometric and knowledge-map analysis
Source: Front Oncol. 2023 Jan 4;12:1071860. doi: 10.3389/fonc.2022.1071860 (PMC9846108; doi:10.3389/fonc.2022.1071860)
Supplement: Supplementary file 1 [file DataSheet_1.pdf]

Supplemental Material

Figure S1

#1:

((((((((((((((((((((((TS=(Hepatocellular Carcinomas)) OR TS=(Carcinomas, Hepatocellular)) OR TS=(Liver Cell Carcinoma, Adult)) OR TS=(Liver Cancer, Adult)) OR TS=(Adult Liver Cancer)) OR TS=(Adult Liver Cancers)) OR TS=(Cancer, Adult Liver)) OR TS=(Cancers, Adult Liver)) OR TS=(Liver Cancers, Adult)) OR TS=(Liver Cell Carcinoma)) OR TS=(Carcinoma, Liver Cell)) OR TS=(Carcinomas, Liver Cell)) OR TS=(Cell Carcinoma, Liver)) OR TS=(Cell Carcinomas, Liver)) OR TS=(Liver Cell Carcinomas)) OR TS=(Hepatocellular Carcinoma)) OR TS=(Hepatoma)) OR TS=(Hepatomas)) OR TS=(HCC)) OR TS=(primary liver carcinoma)) OR TS=(primary liver cancer)) OR TS=(hepatocarcinoma)) OR TS=(hepatocellular carcinoma)

#2:

(((((((((((((((((((((((TS=(hepatic arterial infusion chemotherapy)) OR TS=(hepatic artery infusion chemotherapy)) OR TS=(hepatic arterial infusion)) OR TS=(hepatic artery infusion)) OR TS=(HAIC)) OR TS=(Infusion, Intra-Arterial)) OR TS=(Intra-Arterial Infusion)) OR TS=(Intra-Arterial Infusions)) OR TS=(Infusions, Intraarterial)) OR TS=(Infusion, Intraarterial)) OR TS=(Intraarterial Infusion)) OR TS=(Intraarterial Infusions)) OR TS=(Infusions, Intra Arterial)) OR TS=(Arterial Infusion, Intra)) OR TS=(Arterial Infusions, Intra)) OR TS=(Infusion, Intra Arterial)) OR TS=(Intra Arterial Infusion)) OR TS=(Intra Arterial Infusions)) OR TS=(Infusions, Regional Arterial)) OR TS=(Arterial Infusion, Regional)) OR TS=(Arterial Infusions, Regional)) OR TS=(Infusion, Regional Arterial)) OR TS=(Regional Arterial Infusion)) OR TS=(Regional Arterial Infusions)

Final retrieval:

(((#2 AND #1) NOT TS=(colo*)) NOT TS=(cholangiocarcinoma)) NOT TS=(pancrea*)

Figure S2

Description	Results
MAIN INFORMATION ABOUT DATA	
Timespan	1974:2021
Sources (Journals, Books, etc)	292
Documents	1026
Average years from publication	12.9
Average citations per documents	28.7
Average citations per year per doc	2.468
References	17686
DOCUMENT TYPES	
article	899
article; proceedings paper	43
review	84
DOCUMENT CONTENTS	
Keywords Plus (ID)	1755
Author's Keywords (DE)	1544
AUTHORS	
Authors	4937
Author Appearances	8701
Authors of single-authored documents	14
Authors of multi-authored documents	4923
AUTHORS COLLABORATION	
Single-authored documents	18
Documents per Author	0.208
Authors per Document	4.81
Co-Authors per Documents	8.48
Collaboration Index	4.88

Figure S3

Year	N	MeanTCperArt	MeanTCperYear
1974	1	60	1.25
1975	0	0	0
1976	0	0	0
1977	1	50	1.11111111111111
1978	1	109	2.47727272727273
1979	0	0	0
1980	1	3	0.0714285714285714
1981	0	0	0
1982	0	0	0
1983	0	0	0
1984	0	0	0
1985	0	0	0
1986	1	52	1.44444444444444
1987	1	41	1.17142857142857
1988	1	29	0.852941176470588
1989	2	97.5	2.95454545454545
1990	0	0	0
1991	14	40.1428571428571	1.29493087557604
1992	18	28.5	0.95
1993	17	30.2941176470588	1.0446247464503
1994	27	41.037037037037	1.46560846560847
1995	21	41.8571428571429	1.55026455026455
1996	17	36.4117647058824	1.40045248868778
1997	19	34.5789473684211	1.38315789473684
1998	22	40.3181818181818	1.67992424242424
1999	28	36.9285714285714	1.6055900621118
2000	21	42.1428571428571	1.91558441558442
2001	20	37.7	1.7952380952381
2002	29	61.8620689655172	3.09310344827586
2003	26	34.2692307692308	1.80364372469636
2004	26	37.1153846153846	2.06196581196581
2005	21	37.9523809523809	2.23249299719888
2006	22	58.2272727272727	3.63920454545455
2007	32	41.1875	2.74583333333333
2008	25	30.44	2.17428571428571
2009	27	30.3333333333333	2.33333333333333
2010	46	25.3478260869565	2.11231884057971
2011	45	20.0222222222222	1.82020202020202
2012	56	32.7857142857143	3.27857142857143
2013	46	23.3913043478261	2.59903381642512
2014	41	17.5121951219512	2.1890243902439
2015	54	21.6111111111111	3.08730158730159
2016	40	21.6	3.6
2017	47	12.8085106382979	2.56170212765957
2018	44	59.7954545454545	14.9488636363636
2019	43	10.7441860465116	3.58139534883721
2020	52	5.48076923076923	2.74038461538462
2021	71	2.26760563380282	2.26760563380282

N: Number of articles per year
MeanTCperYear: Yearly average number of times each document has been cited

Figure S4

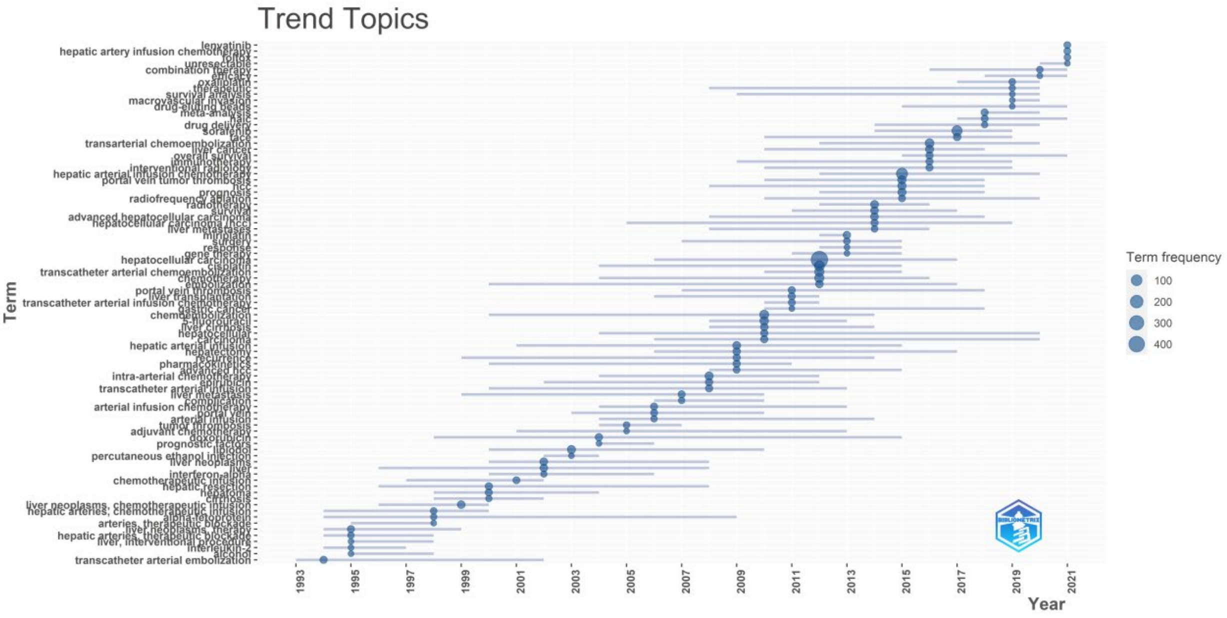

Figure S5

Rank	Journal title	count	h_index	JCR	total citations
1	HEPATO-GASTROENTEROLOGY	49	15	Q1	570
2	JOURNAL OF VASCULAR AND INTERVENTIONAL RADIOLOGY	40	18	Q1	1707
3	HEPATOLOGY RESEARCH	40	17	Q1	792
4	CANCER CHEMOTHERAPY AND PHARMACOLOGY	36	16	Q1	633
5	CARDIOVASCULAR AND INTERVENTIONAL RADIOLOGY	35	15	Q1	1209
6	WORLD JOURNAL OF GASTROENTEROLOGY	29	16	Q1	614
7	CANCER	27	22	Q1	2119
8	ONCOLOGY	25	16	Q1	827
9	ANTICANCER RESEARCH	23	7	Q1	164
10	JOURNAL OF GASTROENTEROLOGY	22	13	Q1	552

Top 10 journals by number of articles.
